# Analyzing power in health systems: the case of medical dominance in India

**DOI:** 10.1186/1753-6561-6-S5-O6

**Published:** 2012-09-28

**Authors:** Kabir Sheikh

**Affiliations:** 1 Public Health Foundation of India, Delhi, India

## Introduction

Power underpins relationships between different actors in health systems and is exercised (or resisted, or subverted) directly by way of coercion and inducement, or indirectly by controlling the ideas and environments that influence other actors to make decisions. Increasingly, abuse and misuse of power have been implicated as key determinants of poor health systems performance in low-income contexts. However the ephemeral nature of power makes it a difficult subject to study, as do the political connotations of such analysis.

## Methods

We present case studies from three separate research projects conducted in varied settings in India. The studies draw from bottom up theories of policy implementation analysis, which help to locate intangible themes such as power in real life events and processes.

i) A qualitative analysis of implementation of global guidelines for HIV testing in five cities (n = 46 medical practitioners, health administrators, policy-planners and donors)

ii) A policy analysis of factors influencing the performance of regulatory institutions for health care, in two states (n = 32 health administrators and regulators)

iii) A health systems ethnography exploring the decision-making processes of doctors working in remote rural areas in Chhattisgarh state (n = 37 medical practitioners)

All three studies utilized qualitative research methodology, including in-depth interviews and document review, and the ‘interpretive’ approach of analysis to understand the experiences of health systems actors.

## Results

i) The analysis of implementation of global guidelines for HIV testing revealed that doctors widely resisted pressures to follow the guidelines, yet could rarely play a role in influencing policy change. A combination of this paradoxical balance of power, conflicts between different actors’ (policy-makers, administrators, practitioners) interpretation of policies, and lack of avenues for the exchange of ideas, contribute to the rift between written policies and field level practices.

ii) The second study describes how public institutions for health care regulation have been subjected to ‘capture’ by medical professional groups that are represented in these institutions. The performance of core functions of these regulatory bodies is frequently subverted or obstructed by the forces embedded within, with the objective of protecting or serving specific vested interests.

iii) The final case study highlights how doctors performing crucial roles in providing health care in remote rural areas face adversity not only from poor working and living conditions, but also in the form of unsupportive administrative structures, unaccountable promotion and transfer policies, and poor access to continued education.

## Discussion

These three studies present a heterogeneous picture of the power of medical professionals in the context of national health systems. While medical professionals continue to hold sway over key administrative and regulatory institutions, this power tends to be directed to the protection of pecuniary and petty political interests, rather than to the upliftment of medical practice. Even as rural doctors struggle to perform under the yoke of unjust administrations, there is evidence that doctors in cities may also be powerless to influence the policies that guide the norms of their practice.

The case studies collectively highlight the crucial role of medical professional power and interests in influencing health systems performance in India, and also demonstrate that a closer appreciation of doctors’ vulnerabilities is necessary in order to confront the problem of medical dominance. At the same time they draw attention to the embeddedness of health systems in society – societal norms, structures and balances of power – and consequently of the necessity of *societal* reforms favouring justice and equity, for improving health systems performance. More evidence on the sociology of health systems is called for, as India moves towards sweeping health sector reforms.

## Competing interests

Author declares that he has no conflict of interest.

## Funding statement

The author declares that the research studies on which this paper is based were funded by the Aga Khan Foundation and University of London (study 1); the Nossal Institute, University of Melbourne (study 2); the World Health Organization and the Government of India (National Rural Health Mission) (study 3).

